# *Gelidium elegans* Regulates the AMPK-PRDM16-UCP-1 Pathway and Has a Synergistic Effect with Orlistat on Obesity-Associated Features in Mice Fed a High-Fat Diet

**DOI:** 10.3390/nu9040342

**Published:** 2017-03-30

**Authors:** Jia Choi, Kui-Jin Kim, Eun-Jeong Koh, Boo-Yong Lee

**Affiliations:** Department of Food Science and Biotechnology, College of Life Science, CHA University, Seongnam, Kyeonggi 463-400, Korea; wldk3176@gmail.com (J.C.); Kuijin.Kim@cha.ac.kr (K.-J.K.); kej763@naver.com (E.-J.K.)

**Keywords:** *Gelidium elegans*, *Gelidium amansii*, high-fat diet-induced obese mice, hyperglycemia, obesity

## Abstract

The incidence of obesity is rising at an alarming rate throughout the world and is becoming a major public health concern with incalculable social and economic costs. *Gelidium elegans *(GENS), also previously known as *Gelidium amansii*, has been shown to exhibit anti-obesity effects. Nevertheless, the mechanism by which GENS is able to do this remains unclear. In the present study, our results showed that GENS prevents high-fat diet (HFD)-induced weight gain through modulation of the adenosine monophosphate-activated protein kinase (AMPK)-PR domain-containing16 (PRDM16)-uncoupling protein-1 (UCP-1) pathway in a mice model. We also found that GENS decreased hyperglycemia in mice that had been fed a HFD compared to corresponding controls. We also assessed the beneficial effect of the combined treatment with GENS and orlistat (a Food and Drug Administration-approved obesity drug) on obesity characteristics in HFD-fed mice. We found that in HFD-fed mice, the combination of GENS and orlistat is associated with more significant weight loss than orlistat treatment alone. Moreover, our results demonstrated a positive synergistic effect of GENS and orlistat on hyperglycemia and plasma triglyceride level in these animals. Thus, we suggest that a combination therapy of GENS and orlistat may positively influence obesity-related health outcomes in a diet-induced obese population.

## 1. Introduction

Obesity is a global health concern that has reached epidemic proportions [1]. Obesity is caused by body fat accumulation in adipose tissue due to abnormalities in energy metabolism. It is associated with a number of metabolic diseases such as diabetes, cardiovascular disease, and low chronic inflammation [2,3]. Although pharmaceutical treatment of obesity costs approximately $ 2 trillion per year [4], the population of obese individuals is rapidly growing worldwide, indicating that prevention or intervention of obesity may be difficult due to the complex multisystem pathophysiology [5]. Clinical guidelines from several countries recommend pharmacological agents such as orlistat for obese patients [6,7]. Orlistat is a Food and Drug Administration (FDA)-approved medicine for the treatment of obesity in the United States. It is an inhibitor of intestinal lipase that decreases dietary fat absorption [8]. 

Although orlistat treatment helps to reduce weight [9,10], it is now generally believed that physical activity and a healthy diet may prevent weight gain and obesity by maintaining energy balance and efficiently burning excessive energy [11]. Increasing consumption of fruits, vegetables, and edible plants and decreasing consumption of high-calorie foods leads to a decrease in weight gain and stabilization of weight in participants [12]. The bioactive compounds present in these food categories that are implicated in obesity prevention include resveratrol, curcumin, conjugated linoleic acid, and omega-3 fatty acids [13,14,15,16]. 

Recent studies have indicated that seaweeds and their derivatives may also have potential therapeutic implications for obesity [17,18]. In particular, *Gelidium elegans *(GENS), previously known as *Gelidium amansii*, has been shown to have nutraceutical activities such as anti-adipogenesis and anti-obesity effects [19,20,21]. However, the mechanism by which GENS is able to achieve its anti-obesity effects remains to be clearly defined. We have previously demonstrated that GENS has the potential to alter adipocyte phenotypes to beige-like adipocytes in vitro [22]. This suggests that GENS may act as an energy expenditure enhancer by stimulating the PR domain-containing16 (PRDM16)/peroxisome proliferator-activated receptor gamma coactivator 1 alpha (PGC1α)/uncoupling protein-1 (UCP-1) pathway in vivo. We therefore aimed to determine the molecular mechanism of GENS and the efficacy of the combination therapy of GENS and orlistat on weight gain, blood biochemistry and gene expression changes in high-fat diet (HFD)-induced obese mice. 

## 2. Materials and Methods

### 2.1. Materials

GENS extract was obtained from NEWTREE Inc. (Seongnam, Kyeonggi, Korea). The composition of GENS extract is described in Table 1. The following antibodies were purchased from Santa Cruz Biotechnology (Dallas, TX, USA): CCAAT/enhancer binding protein alpha (C/EBPα, SC-61), diacylglycerol O-acyltransferase-1 (DGAT-1, SC-32861), fatty acid synthase (FAS, SC-20140), peroxisome proliferator activated receptor gamma (PPARγ, SC-7273), glyceraldehyde 3-phosphate dehydrogenase (GAPDH, SC-25778), sterol regulatory element binding protein-1 (SREBP-1, SC-366), and uncoupling proteins-1 (UCP-1, SC-6529). PR domain-containing16 (PRDM16, ab106410) were obtained from Abcam (Cambridge, MA, USA). Phospho-adenosine monophosphate-activated protein kinase (p-AMPK, CS-2603s), AMPK (CS-2603s), phospho-acetyl-CoA carboxylase (p-ACC, CS-3661s), and ACC (CS-3662s) were purchased from Cell Signaling Technology (Bedford, MA, USA). Orlistat and metformin were purchased from Cayman Chemical Company (Ann Arbor, MI, USA). Glucose and methylcellulose were purchased from Sigma (Sigma, St. Louis, MO, USA). All chemicals and reagents used were of analytical and obtained from commercial sources. 

### 2.2. Animal Husbandry and Maintenance

Male ICR mice (5 weeks old) were purchased form Orient Bio Co. (Gapyeong, Kyeonggi, Korea) and maintained in the animal facility at CHA University, Seongnam, Kyeonggi, Korea. The project was approved by the Institutional Animal Care and Use committee of CHA University (IACUC Approval Number 150071). Male mice were individually housed for 1 week under a 12-h light/dark cycle in temperature (20–24 °C) and humidity (44.5%–51.8%). After a 1-week adaptation period, mice were randomly divided into six groups (*n* = 6 per group). Mice were fed for 7 weeks with either 60% HFD (Central Lab Animal Inc., Seoul, Korea) or NIH-07 rodent chow diet (Zeigler Brothers, Gardners, PA, USA). GENS (50, 200 mg/kg/day) and/or orlistat (20 mg/kg/day) or an equal volume of vehicle (1.0% methylcellulose, HFD and chow diet group) were orally administered to the mice by gavage every day for 7 weeks. During the experiment period, and survival rates were investigated daily. The body weight, food intake, and water consumption were recorded weekly.

### 2.3. Concentration of Blood Glucose

The blood glucose concentration was measured from mice tail vein after 12 h (dark-period) of fasting every week, using a glucose analyzer (GlucoDr, Allmedicus, Kyeonggi, Korea).

### 2.4. Intraperitoneal Glucose Tolerance Test (IPGTT)

Mice were fasted for 12 h (dark-period) before IPGTT experiments, and glucose (1.0 g/kg body weight) was administered by intraperitoneal injection), as previously described [24]. Blood samples were collected from tail vein at 30, 60, and 90 min to assess in vivo glucose clearance. Blood glucose levels were determined immediately using a glucometer (G-Doctor, Allmedicus, Anyang, Korea).

### 2.5. Biochemical Analysis

Mice were sacrificed by CO_2_ asphyxiation and cervical dislocation. Blood collected by direct cardiac puncture in an ethylenediaminetetraacetic acid (EDTA)-coated tube aseptically. Blood was allowed to clot for 1 h at room temperature and then plasma was isolated by centrifuging the blood at 13,000× *g* for 15 min at 4 °C to collect plasma. The plasma samples were collected and stored at −80 °C. Plasma triglycerides (TG) and high-density lipoprotein (HDL)-cholesterol levels were measured by enzymatically commercial kits (Roche, Mannheim, Germany), Plasma insulin levels were measured by enzymatically commercial kit (Wako Pure Chemical. Ltd., Osaka, Japan). The hepatic TG content was determined using a commercially available TG quantification kit (Cayman Chemical Company, Ann Arbor, MI, USA). 

### 2.6. Organ Weight

Mice were euthanized using CO_2_ and cervical dislocation. Heart, lung, kidney, spleen, liver, abdominal fat, and subcutaneous fat were removed and weighed carefully.

### 2.7. Tissue Samples Preparation for Oil Red O

Hepatic tissues were fixed in 4% paraformaldehyde, embedded in using tissue freezing medium, optimum cutting temperature (OCT) (Cell Path Ltd., Newtown, UK). To detect fat deposition in the liver, frozen sections of 6 μm for liver were rinsed with distilled water, stained with 0.18% Oil red O (Sigma, St. Louis, MO, USA) with 60% 2-propanol (Sigma, St. Louis, MO, USA) for 20 min at 37 °C, and then rinsed with distilled water and examined under light microscopy with Nikon Eclipse E600 (Nikon, Tokyo, Japan). 

### 2.8. Western Blot Analysis

For protein extraction, frozen tissues were homogenized in specific lysis buffer (PRO-PREP; iNtRON Biotechnology Inc., Seoul, Korea) containing protease with phosphatase inhibitor cocktail 2 and 3 (Sigma, St. Louis, MO, USA). The lysates were clarified by centrifugation at 12,000× *g* for 20 min at 4 °C. The protein concentration of clarified supernatants was determined by the Bradford assay (Bio Legend, San Diego, CA, USA). Protein samples (30 μg) were separated by sodium dodecyl sulfate polyacrylamide gel electrophores (SDS-PAGE), and transferred onto polyvinylidene fluoride (PVDF) membranes (Bio-Rad, Hercules, CA, USA), as previously described [25]. The membranes were probed with each antibodies and visualized using an enhanced chemiluminescence substrate. The signals were detected with LAS image software (Fuji, New York, NY, USA).

### 2.9. Quantitative Reverse Transcription Polymerase Chain Reaction (Quantitative RT-PCR) Analysis

Mouse liver RNA was extracted by using TRIzol reagent (Invitrogen, Carlsbad, CA, USA). Total RNA was reverse transcribed with the Maxime PCR premix Kit (iNtRON Biotechnology, Seongnam, Korea). Quantitative RT-PCR using SYBR Green 2× master mix kit (m.biotech., Inc., Seoul, Korea) was run in hexaplicate on a CFX96 Touch real-Time PCR Detection System (Bio-Rad, Hercrules, CA, USA). The 18s was used for the relative quantization of the target genes based on the comparative ∆∆ threshold cycle (*C*t) method, as previously described [26]. Primer sequences are shown in Table 2. 

### 2.10. Statistical Analysis

All statistical analyses were performed using the Statistical Package for Social Sciences version 12.0 (SPSS, Chicago, IL, USA). One-way analysis of variance (ANOVA) was used for comparisons among group. Significant differences between the mean values were assessed using Duncan’s test. All values are presented as the mean ± standard deviation (SD) values. The *p*-value in the multiple comparison results (e.g., a, b, c, and d) indicate significant differences among the groups, *p* < 0.05.

## 3. Results

### 3.1. Effect of GENS on Changes in Body Weight, Blood Glucose, Insulin, TG, and Gene Expression in Abdominal White Adipose Tissue in HFD-Fed Mice

**Figure 1:**
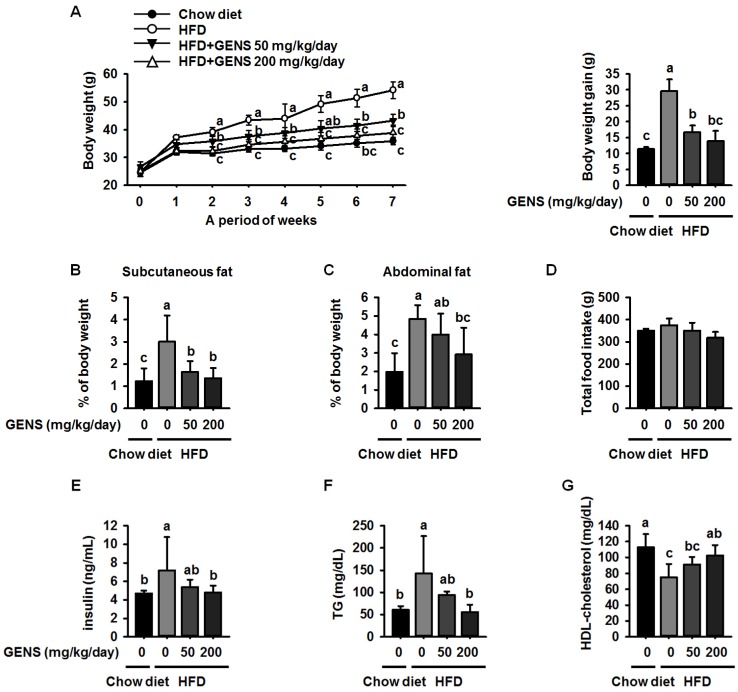
Effect of GENS on body weight change (**A**); subcutaneous fat (**B**); abdominal fat (**C**); total food intake (**D**); serum levels of insulin (**E**); levels of serum triglycerides (TG) (**F**); and high-density lipoprotein (HDL)-cholesterol concentration (**G**) in high-fat diet (HFD)-fed mice. Five-week-old mice were maintained with or without GENS (50 and 200 mg/kg/day) under HFD for 7 weeks. Data are mean ± SD (*n* = 6).

Changes in the body weight of mice fed with chow diet or HFD with the absence or presence of GENS were measured once a week during the experimental period. The introduction of GENS at 50 and 200 mg/kg/day strongly prevented this weight gain and resulted in low amounts of subcutaneous fat and abdominal fat in the mice, as shown in Figure 1A–C. Although the total food intake and average water consumption were not significantly different among the group (Figure 1D and Table 3), the group with HFD alone showed a continuous increase in body weight compared to the chow diet group.

In addition, we observed that the HFD increased the average weight of the liver, subcutaneous fat and abdominal fat compared to the chow diet in mice (Table 4). However, the average weight of the heart, lungs, spleen, and kidney were not affected. Obesity is initially characterized by excess adipose tissue mass and hyperglycemia [27,28].

Finding an effective treatment for hyperglycemia is one of the top priorities in obesity-associated disease research [29]. Moreover, it has been suggested that hyperglycemia in certain types of health conditions is associated with an increased risk of obesity development [30,31].

We therefore investigated whether HFD causes hyperglycemia in our experimental system and whether GENS is able to prevent HFD-mediated hyperglycemia in vivo. As shown in Table 5, the fasting blood glucose level in all group of mice before the treatment was within the normal range (126.0 ± 3.2 mg/dL). We observed that the blood glucose level was significantly increased after 2 weeks of HFD compared to mice fed a chow diet and remained constant for 7 weeks.

In contrast, administration of GENS dramatically prevented this increase in HFD-fed mice. Moreover, hyperglycemia is characterized by an increase in insulin and TG levels in blood serum. Therefore, the level of serum insulin and TG were also measured as shown in Figure 1E,F. The levels of serum insulin and TG in HFD-fed mice were significantly increased as compared to chow diet. However, GENS group dramatically decreased insulin and TG level in blood serum, indicating that GENS may efficiently ameliorate HFD-induced hyperglycemia in these animals. Moreover, it is well known that obesity is frequently associated with low levels of serum HDL-cholesterol [32]. As shown Figure 1G, the HDL-cholesterol in the HFD group were decreased as compared to chow diet. In contrast, administration of GENS significantly increased HDL-cholesterol level in blood serum. Our results thus provide preliminary evidence that GENS has potential as a preventive agent for diet-induced obesity and hyperglycemia. 

### 3.2. Effect of GENS on Adipogenic Factors and IPGTT in HFD-Fed Mice

**Figure 2:**
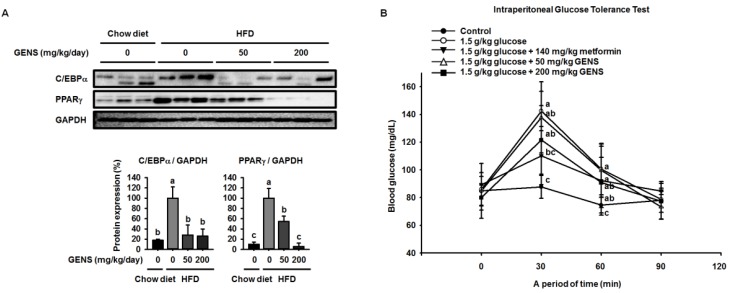
Effect of GENS on key adipogenic factors in HFD-fed mice of white adipose tissue and glucose tolerance. Five-week-old mice were maintained with or without GENS (50 and 200 mg/kg/day) under HFD for 7 weeks. Each parameter was measured by western blot analysis with C/EBPα and PPARγ. The protein expression level was normalized against GAPDH. Protein level was quantified using Image J software (**A**). Five groups of male mice (5 weeks old) were fasted for 12 h (dark-period). After 12-h fasting, mice were administered 1.0 g/kg glucose by intraperitoneal injection. The first group received only Phosphate Buffered Saline (PBS), the second group received 1.0 g/kg glucose, the third group received 1.0 g/kg glucose + 140 mg/kg metformin and the fourth and fifth group received 1.0 g/kg glucose + GENS (50 and 200 mg/kg). Blood glucose levels were measured at the indicated times (0, 30, 60, and 90 min) (**B**). Data are mean ± SD (*n* = 6).

We then analyzed the adipose tissues of HFD-fed mice to confirm how GENS regulated the observed changes. To clarify whether GENS suppress the development of adipose tissue, we analyzed crucial markers of adipogenesis, including C/EBPα and PPARγ [33], by western blotting. As shown in Figure 2A, there was a 5.5-fold increase in the C/EBPα protein level in the adipose tissue of mice fed HFD for 7 weeks compared to that of mice fed a chow diet. There was also a significant elevation of the PPARγ protein expression in the adipose tissue of HFD-fed mice compared to a chow diet. In contrast, GENS at 200 mg/kg/day almost completely suppressed the expression of C/EBPα and PPARγ protein. This result is consistent with recent research, thereby suggesting that the consumption of edible seaweed GENS may result in a lower weight gain through the inhibition of adipose tissue development [19].

Hyperglycemia is characterized by an excessive amount of blood glucose and is often observed in the obese [34,35]. Also, impaired glucose tolerance is a pre-diabetic state of hyperglycemia that is associated with insulin resistance [36]. As shown Figure 2B, the 1.0 g/kg glucose control group reached a glucose level of 142.5 ± 14.2 mg/dL at 30 min. On the other hand, the groups that received 50 or 200 mg/kg GENS had a significantly suppressed rise in blood glucose, with glucose levels of 137.8 ± 25.7 mg/dL and 121.5 ± 24.8 mg/dL at 30 min, respectively. In comparison with the 1.0 g/kg glucose control group, the group that was administered 200 mg/kg GENS significantly decreased their blood glucose levels, by approximately 14.7% at 30 min. At 60 min, the 1.0 g/kg glucose level of the control group reached 100.5 ± 18.6 mg/dL. On the other hand, the groups that received 200 mg/kg GENS had a significantly suppressed the rise in blood glucose, with glucose levels of 90.8 ± 18.0 mg/dL, when compared to the 1.0 g/kg glucose control group at 60 min. 

### 3.3. GENS Represses Hepatic Lipogenesis via the Activation of Thermogenesis-Associated Pathway in HFD-Fed Mice

**Figure 3:**
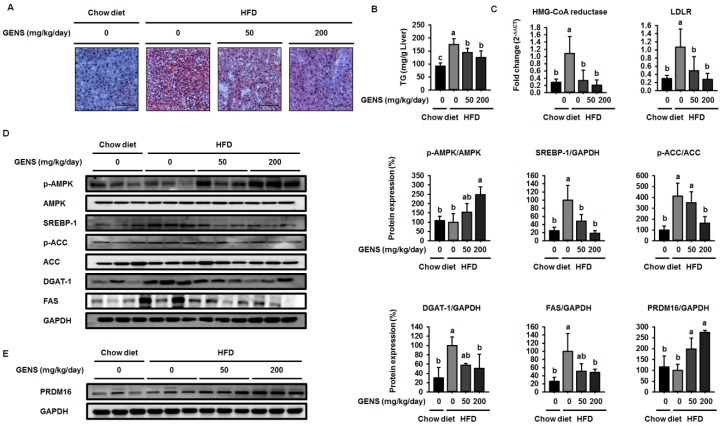
GENS represses hepatic lipogenesis and cholesterol factors in HFD-fed mice. Five-week-old mice were maintained with or without GENS (50 and 200 mg/kg/day) under HFD for 7 weeks. Each parameter was measured by western blot analysis with specific antibodies. The liver sections stained with Oil red O (**A**); Hepatic TG content (**B**); Expression levels of HMG-CoA reductase and LDLR genes were determined by quantitative RT-PCR in liver samples (**C**); p-AMPK, AMPK, SREBP-1, p-ACC, ACC, DGAT-1, and FAS (**D**); PRDM16 (**E**). The protein expression level was normalized against AMPK, ACC, and GAPDH. Protein level was quantified using Image J software. Data are mean ± SD (*n* = 6).

Because previous studies reported that HFD resulted in increases in hepatic lipogenesis and/or non-alcoholic fatty liver [37,38], we also examined the effect of GENS on hepatic lipogenesis in the hepatic tissues of mice. The levels of lipid droplets were evaluated by the combined use of Oil Red O with classic hematoxylin and eosin (H&E) stains in formalin-fixed paraffin-embedded liver tissues. As shown in Figure 3A, Oil Red O staining of liver sections confirmed the abundance of lipid in HFD-fed mice compared to mice fed a chow diet, while GENS prevented hepatic lipogenesis in HFD-fed mice. As shown in Figure 3B, the hepatic TG contents in HFD-fed mice were significantly increased compared to chow diet-fed mice. On the other hand, the group that was administered 50 and 200 mg/kg GENS had significantly suppressed hepatic TG contents, by approximately 28.8% and 26.1%, respectively.

Several studies have demonstrated that SREBP-1 plays an important role in modulating the transcription of genes involved in hepatic lipogenesis, including FAS, ACC, and stearoyl-CoA desaturase1 (SCD1) [39,40]. As shown in Figure 3D, HFD-fed mice markedly enhanced the expression of the SREBP-1 compared to mice fed a chow diet. In contrast, GENS reduced the expression of SREBP-1 in a dose-dependent manner in HFD-fed mice. SREBP-1 regulated transcription of genes such as hydoxymethylglutaryl-CoA (HMG-CoA) reductase and low-density lipoprotein receptor (LDLR) encoding many other enzymes in the cholesterol biosynthetic pathway [41]. We further investigated the effect of GENS on the SREBP-1 downstream target genes that contribute to the intracellular TG synthesis. ACC, DGAT-1, and FAS are downstream target genes of SREBP-1 and play a crucial role in TG synthesis and lipid accumulation [42]. As shown in Figure 3C, the expression of HMG-CoA reductase and LDLR mRNA were notably increased in HFD-fed mice compared to mice fed a chow diet. On the other hand, the GENS group markedly downregulated the expression of HMG-CoA reductase and LDLR mRNA compared to HFD-fed mice. As shown in Figure 3D, the expression levels of the genes DGAT-1 and FAS in HFD-fed mice increased notably compared to mice fed a chow diet. In contrast, the expression of the SREBP-1 downstream target genes ACC, DGAT-1, and FAS were markedly downregulated in the GENS group compared to HFD-fed mice. 

The phosphorylation of AMPK inhibits the expression of SREBP-1 to attenuate hepatic lipogenesis [43]. To determine whether GENS decreased the expression of SREBP-1 through the phosphorylation of AMPK, we examined the AMPK protein by western blot. We observed that HFD slightly decreased the phosphorylation of AMPK compared to chow diet, while GENS stimulated the HFD-induced reduction of AMPK phosphorylation. AMPK is a multiple nutrient sensor and a key energy balance interactor of thermogenic transcription factors including PRDM16 [44,45]. Therefore, our findings indicated that GENS could prevent hepatic lipogenesis via the activation of thermogenesis and energy expenditure. 

PRDM16 is a zinc finger protein that has been proposed to regulate brown adipocyte differentiation, thermogenesis and energy expenditure in adipose tissue and skeletal muscles [36,37]. PRDM16 induces the cellular production of the PGC1α protein, which gives the mitochondria its energy expenditure phenotype [38]. We thus proceeded to investigated if GENS was responsible for the increase in PRDM16 expression. As shown in Figure 3E, HFD with GENS increased the expression of PRDM16 in the mice, whereas HFD alone had no effect on the expression of PRDM16 compared to chow diet. These results suggested that GENS is responsible for the increase in expression of PRDM16, probably via the thermogenesis pathway in brown adipose tissue.

### 3.4. GENS Stimulates the Expression of PRDM16 and UCP-1 Protein in Brown Adipose Tissue and Suppresses Hyperglycemia in HFD-Fed Mice

**Figure 4:**
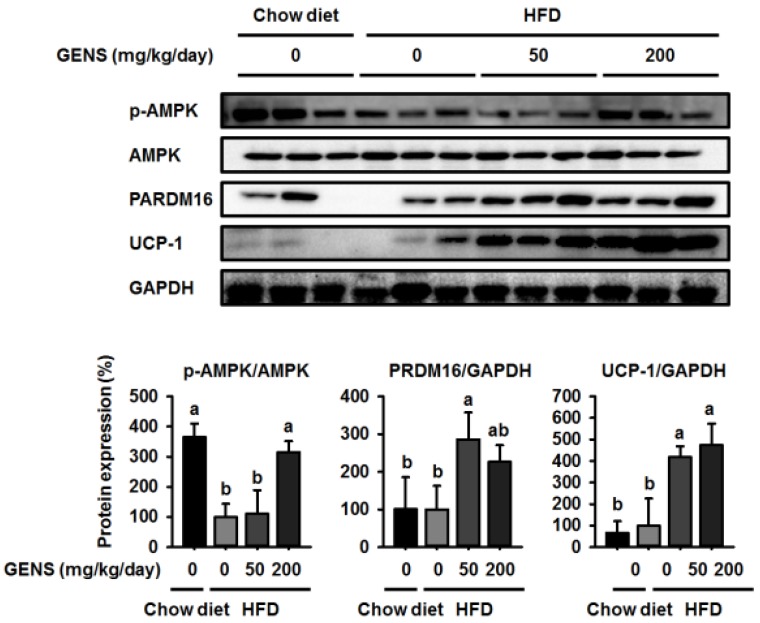
Effect of GENS on energy expenditure protein expression in brown adipose tissue of HFD-fed mice. Five-week-old mice were maintained with or without GENS (50 and 200 mg/kg/day) under HFD for 7 weeks. Each parameter was measured by western blot analysis with p-AMPK, PRDM16, and uncoupling protein-1 (UCP-1). The protein expression level was normalized against AMPK and GAPDH. Protein level was quantified using Image J software. Data are mean ± SD (*n* = 6).

To further assess the role of GENS in the development of brown adipose tissue, we investigated thermogenesis-associated proteins in this tissue. As shown in Figure 4, HFD-fed mice had a lower level of AMPK phosphorylation compared to mice fed a chow diet, while GENS prevented this HFD-induced reduction.

Although there was no difference in the expression of PRDM16 and UCP-1 between mice fed a chow diet and those fed a HFD, we found GENS significantly enhanced the expression of PRDM16 and UCP-1 in brown adipose tissue compared to the corresponding controls. These results suggested that GENS acts as a critical regulator of thermogenesis and the development of brown adipose tissue. 

### 3.5. Effect of Combination of GENS and Orlistat on Body Weight, Organ Weight, Insulin, TG, and HDL-Cholesterol in HFD-Fed Mice

**Figure 5:**
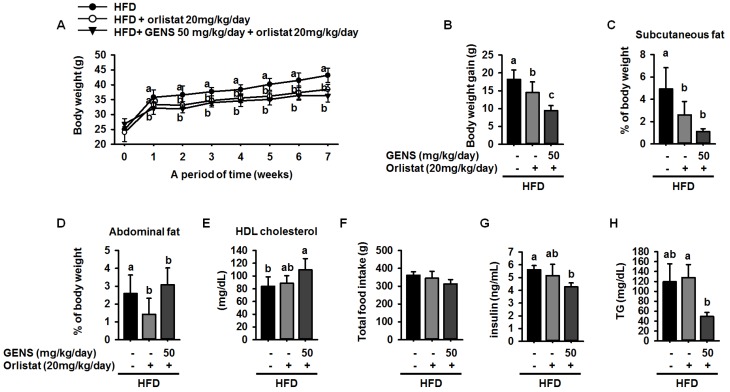
Synergistic effect of GENS and orlistat on weight change (**A**); body weight gain (**B**); subcutaneous fat (**C**); abdominal fat (**D**); HDL-cholesterol concentration (**E**); total food intake (**F**); serum levels of insulin (**G**); and serum levels of TG (**H**) in HFD-fed mice. Five-week-old mice were maintained with or without GENS (50 and 200 mg/kg/day) under HFD for 7 weeks. Data are mean ± SD (*n* = 6).

Our data illustrated a potential mechanism for a thermogenic effect of GENS, suggesting that combination therapy with GENS and orlistat may produce a strong synergistic effect on weight gain prevention in vivo. Based on the above dose response in HFD-fed mice and on previous literature, dosages of 50 mg/kg/day of GENS and 20 mg/kg/day of orlistat were chosen. Orlistat was administered alone or in combination with GENS for 7 weeks in HFD-fed mice. As shown in Figure 5A, orlistat significantly suppressed body weight change compared to HFD-fed mice, and a combination of GENS and orlistat showed a synergistic effect on body weight change. 

In particular, the calculation of total body weight gain revealed that orlistat reduced weight gain by approximately 20.4% compared to HFD-fed mice (Figure 5B). Moreover, GENS and orlistat exhibited better prevention of weight gain in terms of amounts of subcutaneous fat and abdominal fat accumulation in the mice, as shown in Figure 5C,D. In addition, the combination inhibited weight gain compared to HFD-fed mice given orlistat alone. We also observed that serum HDL-cholesterol levels were 84 ± 14.6 mg/dL, 88.6 ± 11.9 mg/dL, and 109.6 ± 17.8 mg/dL in HFD-fed mice, HFD-fed mice treated with orlistat, and HFD-fed treated with GENS + orlistat, respectively. In comparison with HFD-fed mice, mice receiving a combination of GENS and orlistat showed significantly increased serum HDL-cholesterol levels, by approximately 30.5%. (Figure 5E). However, total food intake and average water consumption were not significantly different among the group (Figure 5F and Table 6).

Notably, blood glucose analysis (Table 5) showed consistently that hyperglycemia was initiated at 2 weeks after beginning a HFD and persisted until 7 weeks (Table 7). These data indicated that GENS and orlistat possibly stimulated insulin secretion in the bloodstream. Therefore, we have measured the level of insulin among the group. As shown Figure 5G, the combination of GENS and orlistat significantly suppressed the level of insulin by approximately 24.1% and 8.4% compared to HFD-fed mice or HFD-fed mice with orlistat, suggesting that combination of GENS and orlistat ameliorated impaired insulin homeostasis in HFD-fed mice. 

Although there was an insignificant change in the blood glucose level at 20 mg/kg/day orlistat, combined treatment with GENS and orlistat significantly inhibited the elevation of blood glucose level at 6 and 7 weeks in HFD-fed mice. Furthermore, even though combined treatment with GENS and orlistat did not have any synergistic effect on blood glucose level (Table 7), these mice showed a significant decrease in plasma TG content compared to HFD-fed mice with or without orlistat alone (Figure 5H).

### 3.6. Effect of Combination of GENS and Orlistat on White Adipose Tissue and Hepatic Lipogenesis in HFD-Fed Mice

**Figure 6:**
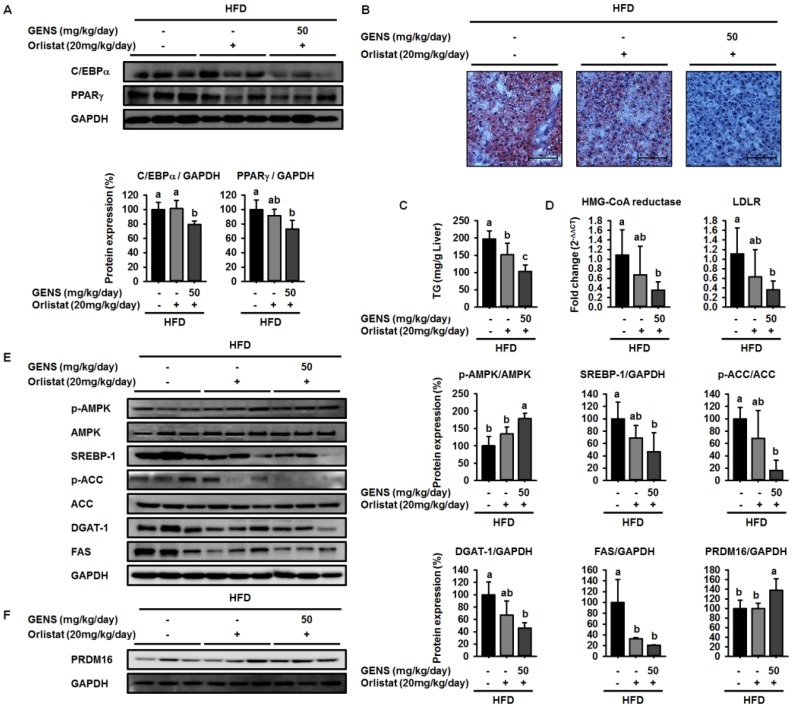
Combination effect of GENS and orlistat on white adipose tissue and hepatic lipogenesis and cholesterol synthesis-associated genes in HFD-fed mice. Five-week-old mice were maintained with or without orlistat (20 mg/kg/day) and GENS (50 mg/kg/day) under HFD for 7 weeks. Each parameter was measured by western blot analysis with C/EBPα and PPARγ (**A**); The liver sections stained with Oil red O (**B**); Hepatic TG content (**C**); Expression levels of HMG-CoA reductase and LDLR genes were determined by quantitative RT-PCR in liver samples (**D**); p-AMPK, AMPK, SREBP-1, p-ACC, ACC, DGAT-1, FAS (**E**) and PRDM16 (**F**). The protein expression level was normalized against AMPK, ACC, and GAPDH. Protein level was quantified using Image J software. Data are mean ± SD (*n* = 6).

To further elucidate the molecular mechanism of the synergistic effect of GENS and orlistat on white adipose tissue and hepatic lipogenesis, we used western blot to evaluate the expression of C/EBPα and PPARγ in white adipose tissue and SREBP-1, AMPK, and PRDM16 in hepatic tissue. We noticed that high levels of C/EBPα and PPARγ were expressed in HFD-fed mice in the presence or absence of orlistat, as shown in Figure 6A. In contrast, combined treatment of GENS and orlistat reduced the expression of C/EBPα and PPARγ compared to the corresponding control. 

To examine whether the combination of GENS and orlistat synergistically inhibited hepatic lipogenesis, we also performed Oil red O staining of formalin-fixed paraffin-embedded liver from HFD-fed mice. As shown in Figure 6B, Oil red O staining revealed that the combination of GENS and orlistat efficiently decreased hepatic lipogenesis. 

In agreement with this observation, in mice fed with HFD, liver weights were 78.6% lower in orlistat-treated mice and 70.9% lower in those receiving a combination of GENS and orlistat compared to mice that were fed a HFD alone (Table 8). Moreover, in mice fed with HFD, liver TG contents were 58.8% lower in those receiving a combination of GENS and orlistat compared to HFD-fed mice (Figure 6C). The combination of GENS and orlistat dramatically decreased the expression of HMG-CoA reductase and LDLR in HFD-fed mice (Figure 6D). In addition, western blot analysis revealed that the combination of GENS and orlistat synergistically increased the phosphorylation of AMPK and decreased the expression of SREBP-1, ACC, DGAT-1, and FAS in HFD-fed mice, as shown in Figure 6E.

**Figure 7:**
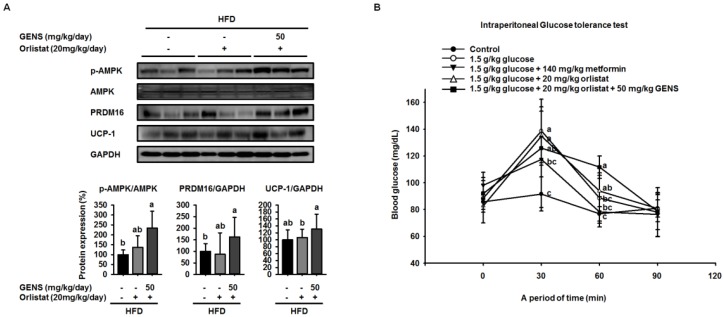
Combination effect of GENS and orlistat on energy expenditure-associated protein expression in brown adipose tissue of HFD-fed mice**. **Five-week-old mice were maintained with or without orlistat (20 mg/kg/day) and GENS (50 mg/kg/day) under HFD for 7 weeks. Protein extracts from brown adipose tissue were assayed for p-AMPK, PRDM16, and UCP-1 by western blot analysis with specific antibodies. The protein expression level was normalized against GAPDH (**A**). Protein level was quantified using Image J software. Five groups of male mice (5 weeks old) were fasted for 12 h (dark-period). After 12-h fasting, mice were administered 1.0 g/kg glucose by intraperitoneal injection. The first group received only PBS, the second group received 1.0 g/kg glucose, third groups received 1.0 g/kg glucose + 140 mg/kg metformin, the fourth group received 1.0 g/kg glucose + 20 mg/kg orlistat and the fifth group received 1.0 g/kg glucose + 20 mg/kg orlistat + 50 mg/kg GENS. Blood glucose levels were measured at the indicated times (0, 30, 60, and 90 min) (**B**). Data are mean ± SD (*n* = 6).

We also found that orlistat alone did not change the expression of PRDM16, but rather the combination of GENS and orlistat significantly induced the expression of PRDM16, thereby indicating that orlistat can contribute to inhibiting lipid metabolism but does not affect thermogenesis (Figure 6F). We sought to further investigate of the effect of combination of GENS and orlistat on thermogenesis-associated proteins in brown adipose tissue by western blot. Consistent with the above observations, we found that orlistat alone induced a statistically insignificant increase in the AMPK phosphorylation, PRDM16, and UCP-1 expression, whereas the combination of GENS and orlistat dramatically elevated AMPK phosphorylation and the expression of PRDM16 and UCP-1 (Figure 7). These results suggested that orlistat plays the role of a negative regulator of lipogenesis, while GENS synergistically acts as an enhancer of thermogenesis and subsequently inhibits the progression of adipogenesis and changes in circulating TG content from hematogenous spread in HFD-fed mice in the presence of orlistat.

To further analyze whether GENS and orlistat plays a role in systemic glucose sensitivity, we performed IPGTT. As shown Figure 7B, the 1.0 g/kg glucose control group reached a glucose level of 138.7 ± 23.7 mg/dL at 30 min. On the other hand, the groups that received combination group (20 mg/kg orlistat + 50 mg/kg GENS) had a significantly suppressed rise in blood glucose, with glucose levels of 125.8 ± 8.9 mg/dL at 30 min. In comparison with the 1.0 g/kg glucose control group, the group that was administered combination group (20 mg/kg orlistat + 50 mg/kg GENS) significantly decreased their blood glucose levels by approximately 9.3% at 30 min. In the current study, our result showed GENS improved glucose homeostasis in vivo. Moreover, the group that was administered 200 mg/kg GENS and combination group (20 mg/kg orlistat + 50 mg/kg GENS) had a significant decrease in blood glucose levels after glucose loading.

## 4. Discussion

Here, we showed that GENS is sufficient to inhibit multiple characteristics of obesity, including weight gain, adipose tissue mass, hepatic lipogenesis, and hyperglycemia, in the HFD-induced obese mouse model. Notably, GENS altered energy metabolism by stimulating the expression of known thermogenesis regulator molecules such as PRDM16 and UCP-1. Moreover, we demonstrated that combined treatment with GENS and orlistat attenuated the plasma TG content and body weight gain in HFD-fed mice. In obesity, excessive energy intake promotes an increase in the storage of lipids in adipose tissue [39], thus resulting in an increase in adipose tissue mass through the formation of new adipocytes and an increase in the size of adipocytes [40]. These events involve cellular and molecular changes including morphological modifications of a number of critical adipogenic transcription factors, such as C/EBPα and PPARγ [28]. HFD can activate the expression of C/EBPα and PPARγ in tissues and cause hyperglycemia resulting from diet-induced early insulin resistance through hematogenous spread [41]. The elevated C/EBPα and PPARγ expression and increased hyperglycemia in obese mice was ameliorated by the intake of fruits, vegetables, and edible seaweed and/or its derivatives. This suggests the natural dietary substances can attenuate characteristics associated with obesity [12,46,47,48].

Our previous study, as well as recent studies, suggested that GENS may exert a protective effect on adipogenesis in adipocytes in vitro [49] and in adipose tissue mass in vivo by regulating C/EBPα and PPARγ proteins [19]. Consistent with previous results, our results showed that GENS decreased the expression of C/EBPα and PPARγ in white adipose tissue in HFD-fed mice. 

Diet-induced hepatic lipogenesis is associated with non-alcohol-induced fatty liver (NAFLD), a disease state that is a crucial factor in insulin resistance and early-stage diabetes [50]. Prior studies have suggested that GENS has the potential to ameliorate hepatic lipid accumulation and plasma TG content in rats that have been fed a high fructose diet [51]. 

However, these studies did not provide molecular mechanisms for how GENS regulates these processes. In this study, we demonstrated that GENS suppressed hepatic lipogenesis through modulation of the AMPK-SREBP-1 pathway in HFD-fed mice. In regulating energy metabolism, AMPK stimulates the interaction between PGC1α and PRDM16 resulting in increased mitochondrial biogenesis, thermogenesis, and energy expenditure [52]. Interestingly, we noticed that in HFD-fed mice treated with GENS, PRDM16 in the liver was significantly increased, indicating that GENS may elevate mitochondrial biogenesis or thermogenesis via activation of the thermogenic factor PRDM16 in these animals.

It has been previously demonstrated that HFD induces hepatic lipogenesis, leading to hyperglycemia [53]. In this study, we revealed that hyperglycemia was induced in HFD-fed mice, whereas GENS significantly prevented HFD-mediated hyperglycemia. Although we could not address the detailed mechanism of the interaction between hepatic lipogenesis and hyperglycemia, we speculate that mitochondrial function such as biogenesis and thermogenesis might be involved in the link in these processes.

We hypothesized that if the anti-obesity effects of GENS were mainly due to the affect thermogenesis, GENS might alter the expression of thermogenesis-associated protein in brown adipose tissue. Thus, we analyzed the expression of thermogenic genes in brown adipose tissue, and our results revealed that levels of the most important thermogenesis-associated proteins AMPK, PRDM16, and UCP-1 were dramatically increased. Previous work has demonstrated that AMPK increases the expression of Sirt1 and enhances mitochondrial biogenesis through the regulation of PGC1α [54]. It is well known that PRDM16 plays a crucial role in controlling the expression of UCP-1 in brown adipocytes [55]. Therefore, we postulated that GENS may stimulate thermogenesis or energy expenditure through the AMPK-PRDM16-UCP-1 pathway in HFD-fed mice. 

Orlistat has been used to promote weight loss and plasma TG content normalization in NAFLD and obesity patients [56,57]. It has also been reported as a specific intestinal lipase that breaks down TG, thus resulting in a decrease in fat absorption [58]. As orlistat treatment can promote the inhibition of dietary fat absorption and fatty acid synthesis, we utilized a different mechanism of activity than GENS to assess whether the combination of low-dose GENS and orlistat affected obesity characteristics in HFD-fed mice. We found that the combination was effective in weight-gain prevention in HFD-fed mice. More importantly, the combined treatment with low-dose GENS and orlistat significantly inhibited the expression of C/EBPα and PPARγ in white adipose tissue. It was reasonable to assume that the beneficial effect of GENS and orlistat on adipose tissue mass in HFD-fed mice was through the inhibition of adipogenesis since there was a significant difference in this variable between the HFD group, the orlistat group, and the low-dose GENS + orlistat group. As the highest level of reduction in hyperglycemia and plasma TG content was achieved with the combination of low-dose GENS and orlistat in HFD-fed mice, this combination seems ideal to reduce obesity features. In addition, we noticed that in these mice, the thermogenic genes AMPK, PRDM16, and UCP-1 were also synergistically activated by the combination treatment. This might indicate that an active UCP-1 protein is a heat generator that in mice contributes to loss of energy in the form of heat. The animal study was performed at 20–24 °C, whereas thermoneutrality for mice is 28–30 °C. Therefore, the UCP-1 protein would be expected to be active in the mice housed under the conditions used in the present study. As the GENS mice had less adipose tissue, both subcutaneous and abdominal, they also have less insulating material. This could mean that these mice had to generate more heat through UCP-1 activation. Obviously, this would contribute to less adipose tissue mass as observed, but the mechanism could be independent of any effect of GENS or orlistat. Under these circumstances, a reduced fat absorption would contribute to less available energy, and hence accentuate the mobilization of fatty acids from white adipose tissue for UCP-1 heat generation. Although the mechanisms of action of GENS and orlistat are distinct, we have provided partial evidence that GENS and orlistat can act synergistically in HFD-fed mice. 

## 5. Conclusions

It is known that the incidence of overweight and obesity is responsible for increased abnormalities in multiple signaling pathways, including adipogenesis, lipolysis, and energy expenditure-associated pathways [59,60], and can cause hyperglycemia and NAFLD. In this study, we provided evidence to suggest that in HFD-fed mice, thermogenesis is activated in response to GENS consumption, possibly through the AMPK-PRDM16-UCP-1 pathway. 

This results in the modulation of hepatic lipogenesis as reflected by altered AMPK-PRDM16-UCP-1 pathway and suppression of white adipose tissue mass expansion. Moreover, we postulated that combination of GENS and orlistat is associated with more significant weight loss than the orlistat alone in HFD-fed mice. In particular, our results demonstrated a synergistic effect between GENS and orlistat on hyperglycemia and hepatic lipogenesis in HFD-fed mice. Therefore, we suggest that combination therapy with GENS and orlistat may positively influence the outcomes related to obesity characteristics in a diet-induced obese population. 

## Figures and Tables

**Table 1:** The composition of *Gelidium elegans* (GENS) extract [23].

Component	GENS Extract
Carbohydrate	47.6%
Crude protein	16.7%
Moisture	5.1%
Crude ash	24.1%
Total polyphenols	8.79 mg per 1 g

**Table 2:** Sequence identification and primers used for quantitative reverse transcription (RT)-PCR analysis of specific messenger RNA.

Gene	Primer Sequence (5′ to 3′)
**HMG-CoA reductase**	Forward GCGACTATGAGCGTGAACAA
	Reverse TGGAGATCATGTGCTGCTTC
**LDLR**	Forward TGTGGAGCTCATCCTCTGTG
	Reverse CACATGGTGTGAGGTTCCTG
**18s**	Forward CCATCCAATCGGTAGTAGCG
	Reverse GTAACCCGTTGAACCCCATT

HMG-CoA: hydoxymethylglutaryl-CoA; LDLR: low-density lipoprotein receptor.

**Table 3:** Effect of supplementation with GENS on water consumption in chow diet and HFD-induced obese mice for 7 weeks.

Group	Water Consumption (mL)						
	1 Week	2 Weeks	3 Weeks	4 Weeks	5 Weeks	6 Weeks	7 Weeks
**Chow diet**	129.5 ± 0.7 ^a^	125.0 ± 0.0 ^a^	117.0 ± 4.2 ^a^	124.5 ± 0.7 ^a^	117.0 ± 9.9	130.5 ± 2.1 ^a^	119.0 ± 11.3
**HFD**	130.0 ± 1.4 ^a^	103.5 ± 6.4 ^ab^	93.5 ± 2.1 ^b^	90.5 ± 2.1 ^b^	77.5 ± 0.7	114.5 ± 5.0 ^a^	118.5 ± 7.9
HFD+GENS 50 *	99.0 ± 7.1 ^b^	106.5 ± 3.5 ^b^	89.0 ± 11.3 ^b^	91.0 ± 0.0 ^b^	97.0 ± 1.4	96.0 ± 7.1 ^b^	104.5 ± 20.5
HFD+GENS 200 *	110.5 ± 0.7 ^b^	95.5 ± 3.5 ^b^	90.0 ± 0.0 ^b^	106.0 ± 4.2 ^a,b^	82.5 ± 15.0	91.0 ± 2.8 ^b^	99.5 ± 0.7

* (mg/kg/day), Data are mean ± SD (*n* = 6).

**Table 4:** Effect of GENS on organ weight in chow diet and HFD-induced obese mice for 7 weeks.

Group Variables	Organ Weight (g)			
	Chow Diet	HFD		
	GENS 0 *	GENS 0 *	GENS 50 *	GENS 200 *
**Liver**	1.6 ± 0.1 ^c^	1.9 ± 0.3 ^a^	1.5 ± 0.2 ^b^	1.5 ± 0.1 ^b,c^
**Subcutaneous fat**	0.7 ± 0.4 ^b^	2.8 ± 0.6 ^a^	1.6 ± 0.5 ^b^	1.3 ± 0.6 ^b^
**Abdominal fat**	0.5 ± 0.2 ^b^	1.6 ± 0.6 ^a^	0.8 ± 0.2 ^b^	0.6 ± 0.3 ^b^
**Heart**	0.3 ± 0.1	0.3 ± 0.1	0.2 ± 0.0	0.2 ± 0.0
**Lung**	0.3 ± 0.0	0.3 ± 0.1	0.3 ± 0.0	0.3 ± 0.0
**Kidney**	0.7 ± 0.1	0.7 ± 0.1	0.7 ± 0.0	0.7 ± 0.0
**Spleen**	0.1 ± 0.0	0.2 ± 0.0	0.1 ± 0.0	0.1 ± 0.0

* (mg/kg/day), Data are mean ± SD (*n* = 6).

**Table 5:** Effect of supplementation with GENS on fasting glucose level in chow diet and HFD-induced obese mice for 7 weeks.

Group	Blood Glucose (mg/dL)						
	1 Week	2 Weeks	3 Weeks	4 Weeks	5 Weeks	6 Weeks	7 Weeks
**Chow diet**	123.0 ± 20.3	108.7 ± 11.1 ^c^	103.3 ± 15.8 ^c^	115.5 ± 16.4 ^c^	110.7 ± 18.3 ^c^	100.5 ± 11.6 ^c^	105.5 ± 13.3 ^c^
**HFD**	121.8 ± 5.1	185.8 ± 28.0 ^a^	172.7 ± 26.5 ^a^	190.2 ± 39.2 ^a^	161.8 ± 10.7 ^a^	167.8 ± 17.6 ^a^	169.5 ± 22.4 ^a^
HFD + GENS 50 *	133.5 ± 12.3	156.0 ± 13.7 ^a,b^	140.2 ± 14.9 ^b^	143.0 ± 12.5 ^b,c^	143.3 ± 15.0 ^a,b^	144.3 ± 13.5 ^a,b^	131.3 ± 18.2 ^b,c^
HFD + GENS 200 *	142.2 ± 39.4	142.7 ± 24.4 ^b^	134.0 ± 14.1 ^b^	149.0 ± 7.0 ^b^	138.8 ± 12.9 ^a,b^	127.7 ± 21.1 ^bc^	139.7 ± 21.6 ^b^

* (mg/kg/day), Data are mean ± SD (*n* = 6).

**Table 6:** Combination effect of GENS and orlistat on water consumption in HFD-fed mice.

Group	Water Consumption (mL)						
	1 Week	2 Weeks	3 Weeks	4 Weeks	5 Weeks	6 Weeks	7 Weeks
**Chow diet**	138.5 ± 13.4	119.5 ± 7.7	112.5 ± 10.6 ^a^	111.0 ± 19.8	105.0 ± 26.8 ^a^	128.5 ± 0.7 ^a^	125.0 ± 2.8
**HFD**	130.0 ± 1.4	108.0 ± 0.0	93.00 ± 2.83 ^b^	105.5 ± 2.1	81.0 ± 4.2 ^b^	116.0 ± 7.0 ^b^	121.5 ± 9.2
HFD + orlistat 20 *	112.5 ± 23.3	113.5 ± 16.2	97.0 ± 9.9 ^b^	108.5 ± 7.6	92.0 ± 11.6 ^b^	101.0 ± 0.0 ^c^	115.0 ± 0.0
HFD + GENS 50 * + orlistat 20 *	121.0 ± 1.41	121.5 ± 0.7	95.0 ± 4.2 ^b^	99.5 ± 2.1	99.5 ± 2.1 ^b^	90.5 ± 3.5 ^c^	118.0 ± 5.6

* (mg/kg/day), Data are mean ± SD (*n* = 6).

**Table 7:** Combination effect of supplementation with GENS and orlistat on fasting glucose level in HFD-induced obese mice for 7 weeks.

Group	Blood Glucose (mg/dL)						
	1 Week	2 Weeks	3 Weeks	4 Weeks	5 Weeks	6 Weeks	7 Weeks
**Chow diet**	125.3 ± 23.9	115.8 ± 25.2 ^b^	118.0 ± 20.0 ^b^	120.0 ± 14.0 ^b^	113.2 ± 7.6 ^b^	108.2 ± 15.0 ^b^	116.5 ± 11.7 ^b^
**HFD**	123.3 ± 12.2	171.2 ± 30.7 ^a^	145.3 ± 31.8 ^a^	136.0 ± 21.5 ^b^	132.0 ± 16.5 ^a,b^	129.2 ± 18.7 ^a,b^	148.2 ± 29.9 ^a^
HFD + orlistat 20 *	122.0 ± 21.2	132.8 ± 31.6 ^a,b^	135.0 ± 16.8 ^a,b^	169.8 ± 24.5 ^a^	146.7 ± 18.2 ^b^	142.2 ± 23.2 ^a^	143.5 ± 18.3 ^a^
HFD + GENS 50 * + orlistat 20 *	116.7 ± 15.9	133.5 ± 34.0 ^a,b^	156.0 ± 15.1 ^a,b^	143.3 ± 24.3 ^a,b^	124.0 ± 27.0 ^a,b^	135.8 ± 33.5 ^a,b^	126.8 ± 7.1 ^a,b^

* (mg/kg/day), Data are mean ± SD (*n* = 6).

**Table 8:** Effect of GENS and/or orlistat on organ weight in HFD-induced obese mice for 7 weeks.

Group Variables	Organ Weight (g)			
	Chow Diet	HFD		
	GENS 0 *	GENS 0 *	Orlistat 20 *	HFD + GENS 50 * + Orlistat 20 *
**Liver**	1.6 ± 0.0 ^b^	2.0 ± 0.2 ^a^	1.6 ± 0.1 ^b^	1.4 ± 0.1 ^c^
**Subcutaneous fat**	0.7 ± 0.3 ^a,b^	1.1 ± 0.6 ^a^	0.9 ± 0.3 ^a,b^	0.5 ± 0.1 ^b^
**Abdominal fat**	1.2 ± 0.6 ^b^	2.0 ± 0.5 ^a^	1.8 ± 0.5 ^a^	1.0 ± 0.3 ^b^
**Heart**	0.2 ± 0.0	0.3 ± 0.1	0.2 ± 0.0	0.3 ± 0.0
**Lung**	0.3 ± 0.0	0.3 ± 0.0	0.3 ± 0.0	0.3 ± 0.0
**Kidney**	0.7 ± 0.1	0.7 ± 0.2	0.7 ± 0.0	0.7 ± 0.1
**Spleen**	0.2 ± 0.0	0.1 ± 0.0	0.2 ± 0.0	0.1 ± 0.0

* (mg/kg/day), Data are mean ± SD (*n* = 6).
